# Iontophoresis Improved Growth Reduction of Invasive Squamous Cell Carcinoma in Topical Photodynamic Therapy

**DOI:** 10.1371/journal.pone.0145922

**Published:** 2016-01-11

**Authors:** Camila Nunes Lemos, Joel Gonçalves de Souza, Patrícia Sper Simão, Renata Fonseca Vianna Lopez

**Affiliations:** School of Pharmaceutical Sciences of Ribeirão Preto, University of São Paulo, Ribeirão Preto, São Paulo, Brazil; Massachusetts General Hospital, UNITED STATES

## Abstract

This study examined the potential of iontophoresis in topical photodynamic therapy (PDT) of human invasive squamous cells carcinomas (SCC). SCC was induced in nude BALB/c mice by subcutaneous injection of A431 cells. Tumor penetration and distribution of the photosensitizer tetrasulfonated zinc phthalocyanine (ZnPcS_4_) was investigated after 10 and 30 min of *in vivo* iontophoresis of a gel containing ZnPcS_4_. PDT was performed immediately after iontophoresis using laser at 660 nm with a dose of irradiation of 100 J/cm^2^ and irradiance of 48 mW/cm^2^ while tumor growth was measured for 30 days. Iontophoresis increased ZnPcS_4_ penetration into tumors by 6-fold after 30 min when compared with passive delivery. Confocal microscopy analysis showed that ZnPcS_4_ was homogeneous distributed within deep regions of the tumor after iontophoresis. Irradiation of the tumors immediately after iontophoresis showed reduction in tumor size by more than 2-fold when compared to non-treated tumors. Iontophoretic-PDT treated tumors presented large areas of necrosis. The study concluded that iontophoretic delivery of photosensitizers could be a valuable strategy for topical PDT of invasive SCC.

## Introduction

Squamous cell carcinoma (SCC) is a cancer of epithelial origin that begins in the squamous cells. The major head and neck cancers and 20% of skin cancers are SCC [1–3]. Conventional SCC treatments involve the combination of surgery, radiation and chemotherapy. Besides these treatments mentioned above, photodynamic therapy (PDT) has provided satisfactory results in the treatment of skin cancers, however, its use in the treatment of nodular and invasive SCC has been avoided [4].

PDT involves the intravenous administration of a photosensitizer with affinity for cancer cells and the subsequent exposure of these cells to light at specific wavelengths; when the photosensitizer absorbs photons, a photochemical reaction is started, resulting in the production of reactive oxygen species that are responsible for the death of cancer cells through a complex biochemical cascade reaction [5].

In the treatment of skin cancers by PDT, the photosensitizer can be topically administered; this provides a targeted drug release to the cancer cells, thereby reducing side effects associated with systemic administration of the photosensitizer. However, skin is a powerful barrier that prevents drug penetration. Therefore, topical PDT is usually achieved by the administration of photosensitizer precursors, in particular 5-aminolaevulinic acid (ALA) or its derivatives [6]. ALA penetrates the skin, but needs to be converted into protoporphyrin IX, an endogenous photosensitizer, before light irradiation; thus, requiring an incubation period. Moreover, conversion of ALA into the photosensitizer depends on skin metabolism [7] and may be poorly converted in some cell tumors type [8].

Currently, topical PDT using ALA derivatives has been approved for use in Europe for the treatment of non-hyperkeratotic actinic keratosis, squamous cell carcinoma *in-situ* (Bowen’s disease), in addition to superficial and nodular basal cell carcinomas through the application of red-light irradiation after drug penetration [6]. However, its use is not recommended for nodular and invasive SCC due to the low ability of undifferentiated keratinocytes to synthesize protoporphyrin IX after the prodrug application and the poor penetration of the photosensitizer precursors into malignant cells located deeper in the dermis [4].

To increase drug penetration into the skin, many physical methods have been investigated, including the use of iontophoresis [5, 9,10], low frequency ultrasound [11,12], microneedles [13] and electroporation [14]. This paper focuses on the use of iontophoresis as an effective method to improve photosensitizer penetration into the skin for the treatment of invasive SCC.

Iontophoresis involves the application of a low intensity electric current in order to improve the permeation of charged and neutral molecules through different biological barriers such as the skin [15]. In iontophoresis, a constant electric current, not higher than 0.5 mA/cm^2^ is applied through an electrolytic formulation by using a positive (anode) and a negative (cathode) electrode. There are two main mechanisms responsible for increasing drug transport under iontophoretic delivery: electromigration and electrosmosis [16]. The contribution of each mechanism on the amount of drug transported depends on the physicochemical properties of the drug. Overall, electromigration is responsible for increasing transport of charged substances while electrosmosis improves the permeation of neutral substances and macromolecules [9]. It has been shown that the amount of drug transported through the skin by iontophoresis is proportional to the intensity of the electric current applied and the time of application [17]. Moreover, modifications in the formulation can also modulate drug skin penetration and distribution by iontophoresis [18,19].

**Fig 1:**
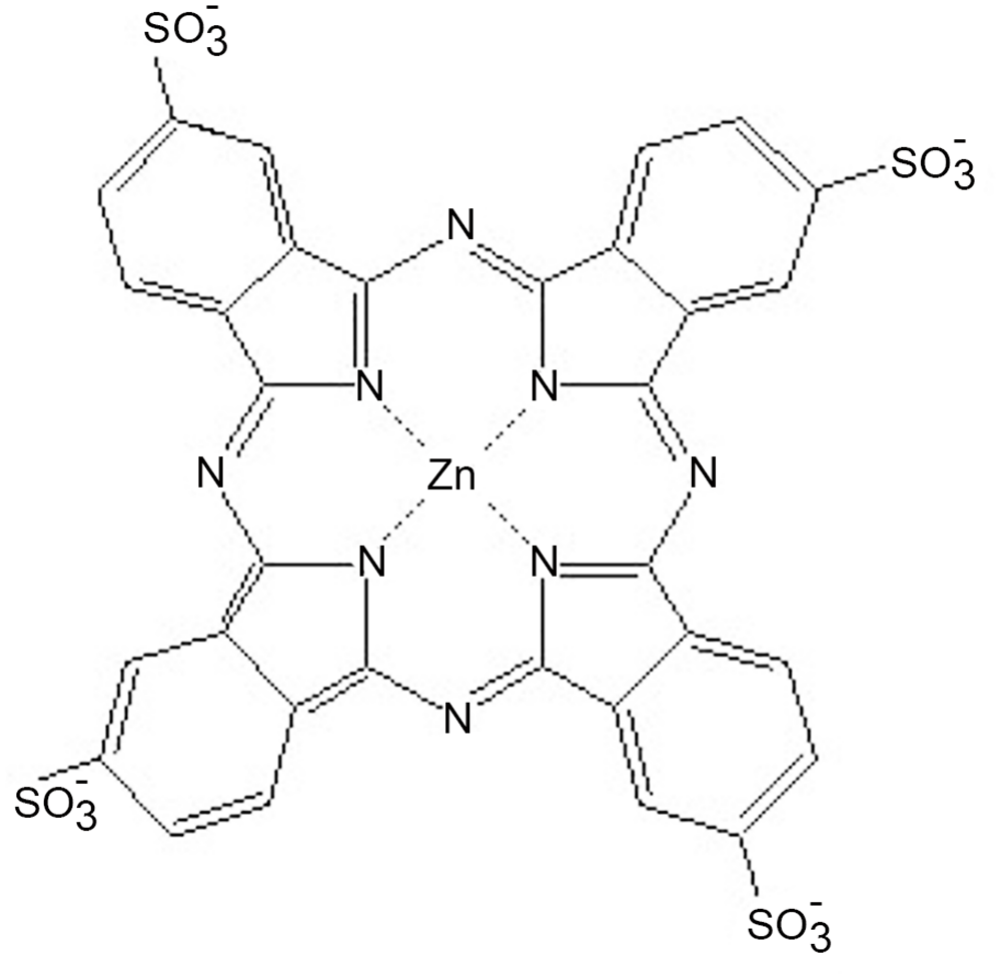
Chemical structure of ZnPcS_4_.

The use of iontophoresis for the improvement of photosensitizer’s [5, 7] or its precursor’s [20–23] penetration into the skin has been demonstrated *in vitro* and *in vivo* using healthy skin. For instance, we have previously showed that *in vitro* iontophoresis of a second-generation photosensitizer, tetrasulfonated zinc phthalocyanine (ZnPcS_4_) (Fig 1) was able to transport significant amounts of the drug to the viable epidermis, while passive drug delivery was irrelevant in 6 h administration [5, 7]. *In vivo* experiments in healthy rats confirmed the ability of iontophoresis to transport large amounts of ZnPcS_4_ to deep skin layers even for short application periods of 15 min [5].

Although the results obtained after iontophoretic delivery of photosensitizers into healthy skin are promising in PDT, the influence of iontophoresis in the penetration and distribution of a photosensitizer in SCC has never been demonstrated. The tumor can hamper electric current passage and drug distribution throughout the malignant cells, thus decreasing PDT efficacy. Therefore, the aim of this work is to evaluate the benefit of iontophoresis in topical PDT of ZnPcS_4_ in human SCC xenografted nude BALB/c mice.

## Experimental section

### 2.1 Chemicals

ZnPcS_4_ was purchased from Frontier Scientific (Logan, Utah, USA), Ag-wire (99.99%, + = 1.5 mm) and AgCl (99.99%) from Sigma-Aldrich (Steinheim, North Rhine-Westphalia, Germany), dimethyl sulfoxide (DMSO) from Vetec (Rio de Janeiro, Rio de Janeiro, Brazil), 4-(2-hydroxyethyl)-1-piperazineethanesulfonic acid (HEPES) from J.T. Baker (Phillipsburg, New Jersey, USA) and hydroxyethylcellulose (HEC) from Galena (Campinas, São Paulo, Brazil). Deionized water (Milli-Q Millipore Simplicity 185, Bedford, MA, USA) was used to prepare all solutions.

### 2.2 Formulation

A non-ionic gel composed of HEC was prepared by weighing 1.5 g of HEC and 5 g of propylene glycol followed by dispersion in 100 g of deionized water. This dispersion was heated and stirred until the formation of the hydrogel. Then, 10 mg of ZnPcS_4_ was dissolved in a minimum amount of water and incorporated into 10 g of the gel after cooling. The pH of the formulation was thereafter adjusted to 5.5 using 1 M NaOH.

### 2.3 Animals

Immunosuppressed (nude) female BALB/c mice, 12–16 weeks (20–25 g average body mass) were purchased from IPEN/CNEN/SP (São Paulo, Brasil). All animal experimental protocols and procedures were approved by the University of São Paulo Animal Care and Use Committee (Authorization number: 08.1.980.53.5 and 11.1.727.53.1) which is in accordance with the National Institutes of Health (NIH) Guidelines for the Care and Use of Laboratory Animals. The animals were kept in a SPF (Specific Pathogen Free) environment, in sterile cages (454 cm^2^, 2 animals per cage), exposed to daily 12:12 h light-dark cycles and natural moisture. The temperature was kept between 24 and 28°C. Before the experiments, the animals were anesthetized by intraperitoneal injection of a mixture of 10% ketamine and 2% xylazine (1:1) at a dose of 100 uL/100 mg body weight. During the experiments, the animals were handled under a laminar flow hood using aseptic techniques.

### 2.4 Tumor induction

Human SCC (A431) cells were obtained from the American Type Culture Collection (Rockville, MD). The cells were cultured in Dulbecco’s Modified Eagle’s Medium (DMEM, pH 7.2–7.4) (Gibco, Grand Island, USA) containing 10% (v/v) of heat-inactivated fetal bovine serum (Gibco, Grand Island, USA), 10,000 U/mL of G penicillin and 10,000 g/mL of ciprofloxacin at 37°C with 5% CO_2_ and a humidified atmosphere.

The tumor was induced by subcutaneous injection of saline containing 2 x 10^6^ SCC cells in the dorsal region of the mice. Tumor growth was measured using a caliper while treatments started 10 days after tumor induction, when the solid tumors reached a volume of about 200 ± 80 mm^3^.

### 2.5 Application time of iontophoresis

To evaluate the effect of iontophoresis on the amount of ZnPcS_4_ retained in the tumors, an open plastic chamber was attached to the animals’ tumor with silicone. Afterwards, 1 g of HEC gel containing 0.1% of ZnPcS_4_ was added inside the chamber. The negative electrode (AgCl) was immersed into the gel, while a counter electrode patch (Iomed^®^, Salt Lake City, Utah, USA) was fixed on the tail of the mouse. Cathodal iontophoresis was performed for 10 min (n = 3) and 30 min (n = 3) by applying a constant electric current of 0.5 mA/cm^2^ using Phoresor^®^ Auto II, Model N°. PM850 (Iomed^®^,Salt Lake City, Utah, USA). A statistical power analysis was performed to define the sample size of animals [24]. Data evaluation was performed according to a confidence interval (bilateral) of 95% and 70% of statistical power.

Immediately after the iontophoresis, tumors were excised and added to a conical tube containing 3 mL of DMSO. All content was homogenized using a tissue homogenizer (IKA T-25 Ultra-turrax, Baden-Württemberg, Germany) at 17,500 rpm for 1 min, centrifuged for 10 min at 6163 g and the supernatant filtered. The amount of ZnPcS_4_ in the sample was quantified by UV/Vis spectrophotometry at 687 nm as previously described [6].

### 2.6 ZnPcS_4_ distribution in the tumor

Animals (n = 5) were treated with the gel containing ZnPcS_4_ for 30 min by cathodal iontophoresis (0.5 mA/cm^2^). Another group (n = 5) was exposed to the ZnPcS_4_ gel without electric current application (passive experiments). A third group (n = 5) did not receive any treatment (negative control). After the treatment, the tumors were excised and immediately frozen using Tissue Tek solution (O.C.T. Compound) at -20°C in order to preserve the fluorescence of the drug. Cryo-sections of 30 μm thickness perpendicular to the surface of the skin were obtained And the distribution of ZnPcS_4_ fluorescence was examined using a Confocal laser microscope (Leica TCS SP5, Mannheim, Germany)) employing a HeNe laser at 633 nm for excitation and an emission band at 640–800 nm.

### 2.7 Tumor regression by PDT

**Fig 2:**
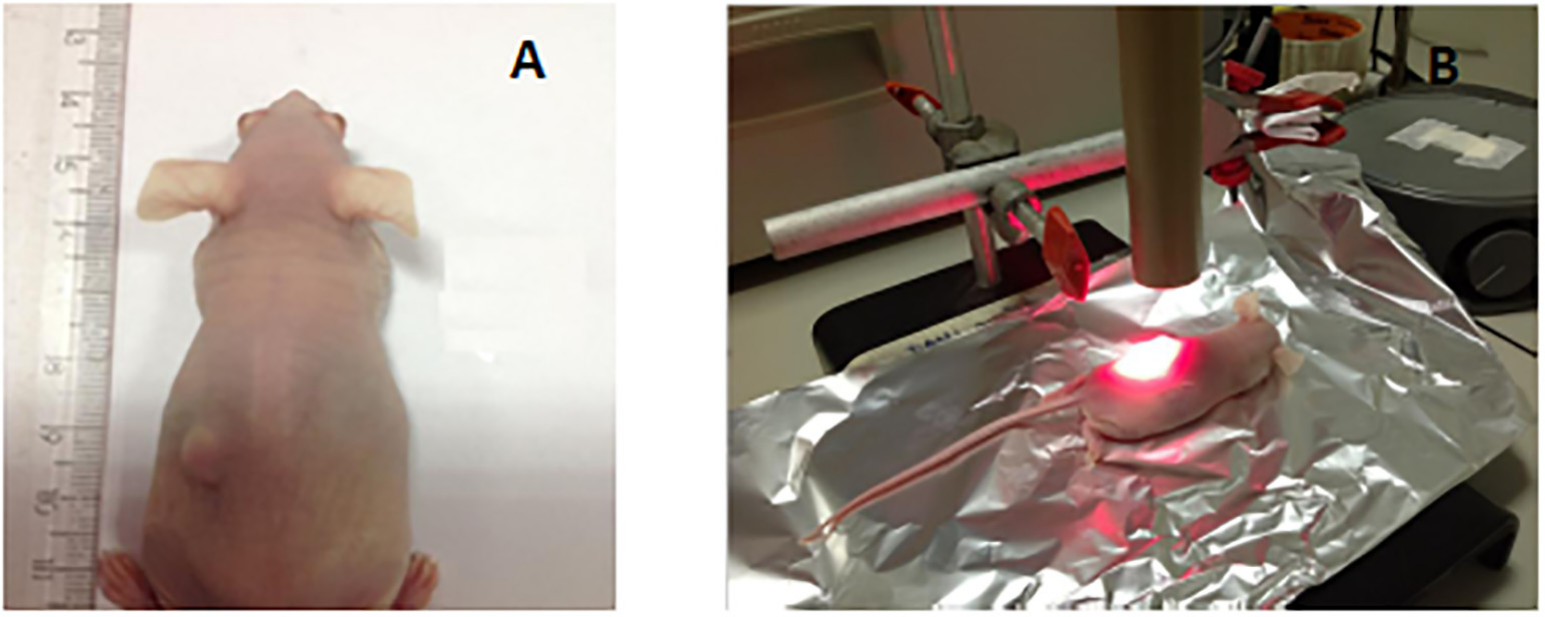
PDT treatment after iontophoresis. A) Subcutaneous xenograft tumor of human SCC (A431) in nude BALB/c mouse. B) Irradiation using a laser light (λ = 660 nm) with a total energy of 100 J/cm^2^ at a distance of 5.0 cm from the tumor surface.

A group of 5 animals were used in the evaluation of tumor regression. A statistical power analysis was performed to define the sample size of animals [24]. Data evaluation was performed according to a confidence interval (bilateral) of 95% and 70% of statistical power. The treatment was performed in two stages. In the first stage, iontophoresis of ZnPcS_4_ gel was performed for 30 min with an electrical current of 0.5 mA/cm^2^. Immediately after iontophoresis, each animal was exposed to the PDT treatment using laser light (λ = 660 nm) with a distance of 5 cm between the tumor and laser probe. In order to standardize the intensity of light reaching the surface of tumor, light intensity was measured with a radiometer (Coherent Fieldmax II Top radiometer, Portland, USA). After standardization, a dose of 100 J/ cm^2^ and irradiance of 48 mW/cm^2^ was applied for 35 min (Fig 2). The second stage of PDT treatment was performed 6 days later using the same light dose (100 J/cm^2^) after iontophoresis treatment. The tumors were photographed (Panasonic Lumix 16 mega pixels, automatic mode, Japan) and their dimensions were measured for 30 days after the last light exposure using a caliper and its volume calculated using the tumor’s diameter.

## Results and Discussion

### 3.1 Formulation

The non-ionic HEC gel at pH 5.5, without any other ions except the ZnPcS_4_ molecule was chosen for *in vivo* iontophoretic experiments. The gel presented an adequate pH and viscosity, thereby preventing its draining during application. It is important to point out that at pH 5.5, ZnPcS_4_ molecules are negatively charged, preventing their aggregation and improving their skin penetration by electromigration [5]. We have previously demonstrated that in the absence of NaCl, cathodal iontophoresis increased about 5-fold the amount of ZnPcS_4_ that penetrated into the viable epidermis in comparison to the same formulation containing NaCl [5]. The decrease in ZnPcS_4_^4-^ penetration by cathodal iontophoresis due to NaCl may be explained by the competition between the ion chloride and ZnPcS_4_^4-^ for electric current transport [25], thereby decreasing the efficiency in drug delivery. Therefore, NaCl was not added to the formulation; however, the passage of a constant electric current during the 30 min experiment was confirmed by monitoring the current and voltage during all the study [7].

### 3.2 Iontophoresis application time

*In vivo* experiments were performed by xenografting Nude BALB/c mice with human SCC cell line A431.

Table 1 shows the amount of ZnPcS_4_ recovered from the tumor after different periods of passive and iontophoretic administration.

**Table 1:** Amount of ZnPcS_4_ recovered from tumor after passive and iontophoretic administration.

Application Time (min)	ZnPcS_4_ after treatment (ng/mg)	
	Passive	Iontophoresis
10	-	6.13 (3.19)
30	1.49 (0.12)	15.21 (8.64)*^,^ ^#^

Data are presented as mean (SD) (n = 3). Unpaired t-test (p ≤ 0.05).

(*) is the statistical difference between drug delivered for 10 and 30 min of iontophoresis.

(^#^) represents the statistical difference between passive and iontophoretic delivery.

(-) means that the results were below the quantification limit of the method (< 0.25 μg/mL) (5).

Table 1 shows that ZnPcS_4_ could not be quantified in tumor after 10 min of passive application but 1.5 ng of the drug penetrated per mg of tumor after 30 min. Iontophoresis significantly improved ZnPcS_4_ tumor penetration/retention by more than 10-fold after 30 min application. Increase in electric current application time from 10 to 30 min increased the amount of drug recovered from the tumor by 2.5-fold.

**Fig 3:**
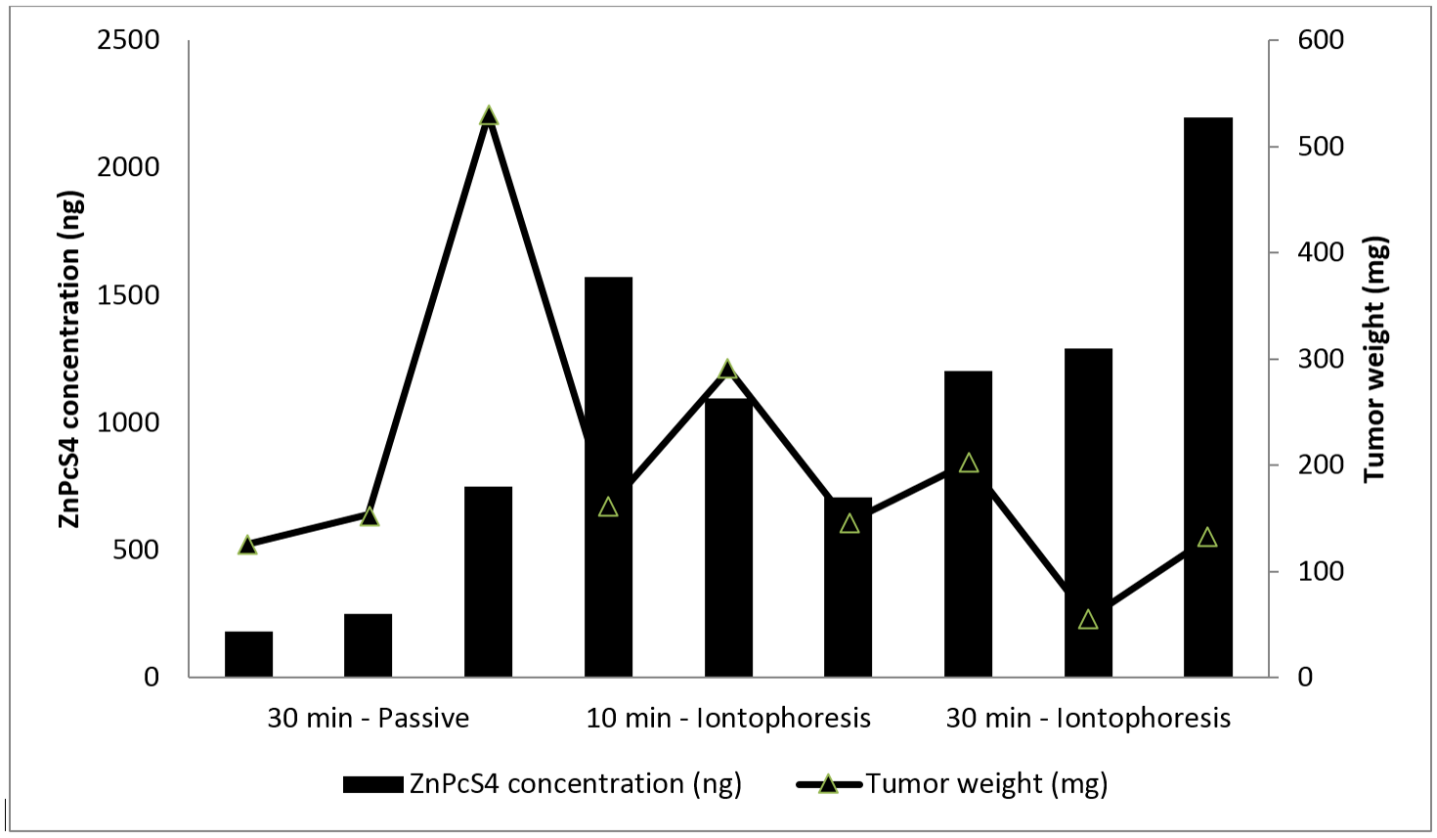
Amount of ZnPcS_4_ recovered from tumor with a specific mass after passive or iontophoretic treatment. The column refers to ZnPcS_4_ concentration and row relates to tumors weight.

It is worth pointing out that the mass of the tumors varied significantly among the experiments (204±137 mg). The application of cells to tumor induction was performed manually, and although the amount injected was the same, the tumors can grow differently due to the particular metabolism of the animals [13], reaching a different final mass after 10 days (when the treatment started). Fig 3 shows the mass of the tumor and the amount of ZnPcS_4_ recovered from each tumor sample after each specific treatment.

After passive experiment, the amount of ZnPcS_4_ recovered from the tumors increased with tumor mass possibly because of the larger area for drug diffusion. After iontophoresis, however, it was not possible to establish a direct correlation between the tumors area and the amount of drug that penetrated (Fig 3). In this case, drug’s penetration is influenced, besides the tumor area, by the applied electric current (I) and the ion transport number (t_x_) (Eq 1) [14].

$$J_{x}^{ER}=\frac{1}{z_{x}AF}t_{x}I$$ (1)

Where J^ER^_x_ represents the electromigration transport of ion x, A is the area of transport through the skin, z_x_ is the charge of ion x, and F is the Faraday constant (96,500 C/mol).

The transport number (t_x_) is defined as the fraction of the total current that is carried by a specific ion [26]. It depends on the physicochemical properties of the ion, such as the charge, mobility and concentration of the drug, and the physical and chemical properties of the other charged molecules present in the system. Therefore, all the ions present in the iontophoretic system compete among one another for the current transport. It means that the presence of other ions can reduce the influx of the drug, consequently, its tumor permeation [27]. In the formulation applied over the tumor, the only ion present was ZnPcS_4_^4-^; however, endogenous ions certainly competed with drug ions and thus decrease the electrotransport of drug into the tumor. The percentage of drug recovered from the tumors was 0.11±0.04% and 0.15±0.05% of the total drug amount applied over the tumor (1 mg) after 10 min and 30 min iontophoresis, respectively. In general, 30-min iontophoresis increased by 1.5-fold ZnPcS_4_ electrotransport when compared to 10-min application. However, increases in drug concentration was not suppose to increase its iontophoretic penetration because, even in the absence of competitive ions in the formulation, the percentage of drug transported into the tumor was smaller than 0.2%. It is possible to suggest therefore, that smaller drug concentration can be used in the formulation resulting in a similar drug penetration, increasing the efficiency of the iontophoretic system.

Note that these experiments were performed using only 3 animals per group in accordance with the recommendation of the "Brazilian Guidelines for the Care and Animal Use for Scientific Purposes and Teaching—DBCA"—2013 and the “University of São Paulo Animal Care and Use Committee” so as to reduce the number of animals in research. Because the amounts of drug recovered after the experiments were reliable and reproducible, we considered that this number of animals were adequate for the purpose of this experimental protocol, which was to establish an application time that makes it possible to compare ZnPcS_4_ penetration in the SCC after passive and iontophoretic experiments. Therefore, further experiments were performed by applying the ZnPcS_4_ gel over the tumor for 30 min by passive or iontophoretic administration.

### 3.3 ZnPcS_4_ tumor distribution

**Fig 4:**
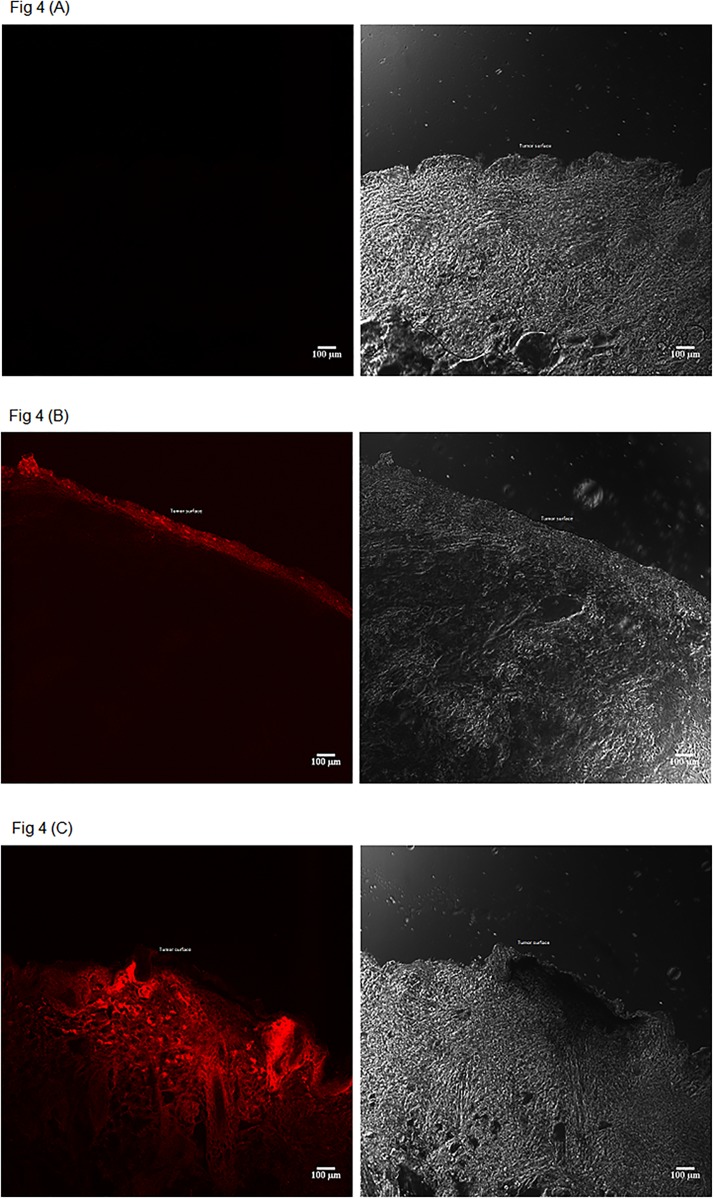
Tumor surface photomicrographs of fluorescence (red) emitted by ZnPcS_4_ in tumors topically treated for 30 min. (A) untreated skin (B) passive application, (C) Cathodal iontophoresis (0.5 mA/cm^2^). The tumor samples were photomicrographed using a 40x objective excitation at 633 nm and detection in the range of 640–800 nm. A non-treated tumor was used as negative control to configure the microscope settings and to eliminate any possible autofluorescence signal from the tumor. All the images were captured using the same parameter. Brightness/contrast enhancement was applied to images B and C using ImageJ software (National Institutes of Health, Bethesda, MD). Bar = 100 μm.

Drug distribution homogeneity and its high concentration within the tumor are important factors that make topical PDT an effective therapeutic tool [28]. Because distribution studies were only qualitative, 5 animals were used after 30-min of passive and iontophoretic experiments to be sure that the high amount of drug recovered from the tumor after iontophoresis (Table 1) was not confined at superficial tumor region but spread all over the tumor mass. Fig 4 shows the photosensitizer distribution in cross-sections of representative tumors after 30 min of passive and iontophoretic application of 0.1% ZnPcS_4_ *in vivo*.

As shown in Fig 4, ZnPcS_4_ did not penetrate passively into the deeper regions of the tumor since drug characteristic fluorescence was observed only on the skin surface. On the other hand, a high fluorescence was observed after cathodic iontophoresis in the surface and the deeper regions of the tumor, suggesting a high ZnPcS_4_ penetration with a homogeneous distribution after 30 min.

The result shows that iontophoresis and its application time influenced the penetration/distribution of the drug into the tumor. Such considerations have high impact on the therapy of skin cancer since the amount of drug accumulated in the tumor is of great importance in the efficiency of PDT [5].

### 3.4 Evaluation of tumor regression after PDT

In order to evaluate the effect of iontophoresis on tumor regression, iontophoresis was applied for 30 min in a group of 5 animals. Experiments without the application of iontophoresis (passive) were not performed because the preliminary studies clearly demonstrated low amounts of ZnPcS_4_ in the tumor under this condition (Table 1). Furthermore, confocal microscopy studies (Fig 4B) showed that ZnPcS_4_ accumulated only on the surface of the tumor when applied passively. The positive control was a group of 3 animals, which did not receive any kind of treatment after tumor induction. The sample size of the control (n = 3) was smaller than the treated group (n = 5) because it is known from the literature that human A431 SCC cells grow exponentially after induction in nude BALB/c mice [29]. Therefore, in an attempt to reduce the number of experimental animals, control sample size was reduced from 5 to 3 animals. Experiments with iontophoresis and sensitizer in the absence of light were not performed at this moment because it is known that phthalocyanines, including ZnPcS_4_, are not cytotoxic in the absence of light in different culture cells [10–12]. For instance, minimal effects were observed in dark conditions in SiHa cells at the high concentration of 2.5 mM [30]. In our study, only 1 mM of ZnPcS_4_ (MM = 898.15 g/mol; 0.1%) was applied over the skin and about 15 ng/mg (see Table 1) reached the tumor. Therefore, any form of cytotoxic effect of ZnPcS_4_ is not expected at this concentration in the dark.

**Fig 5:**
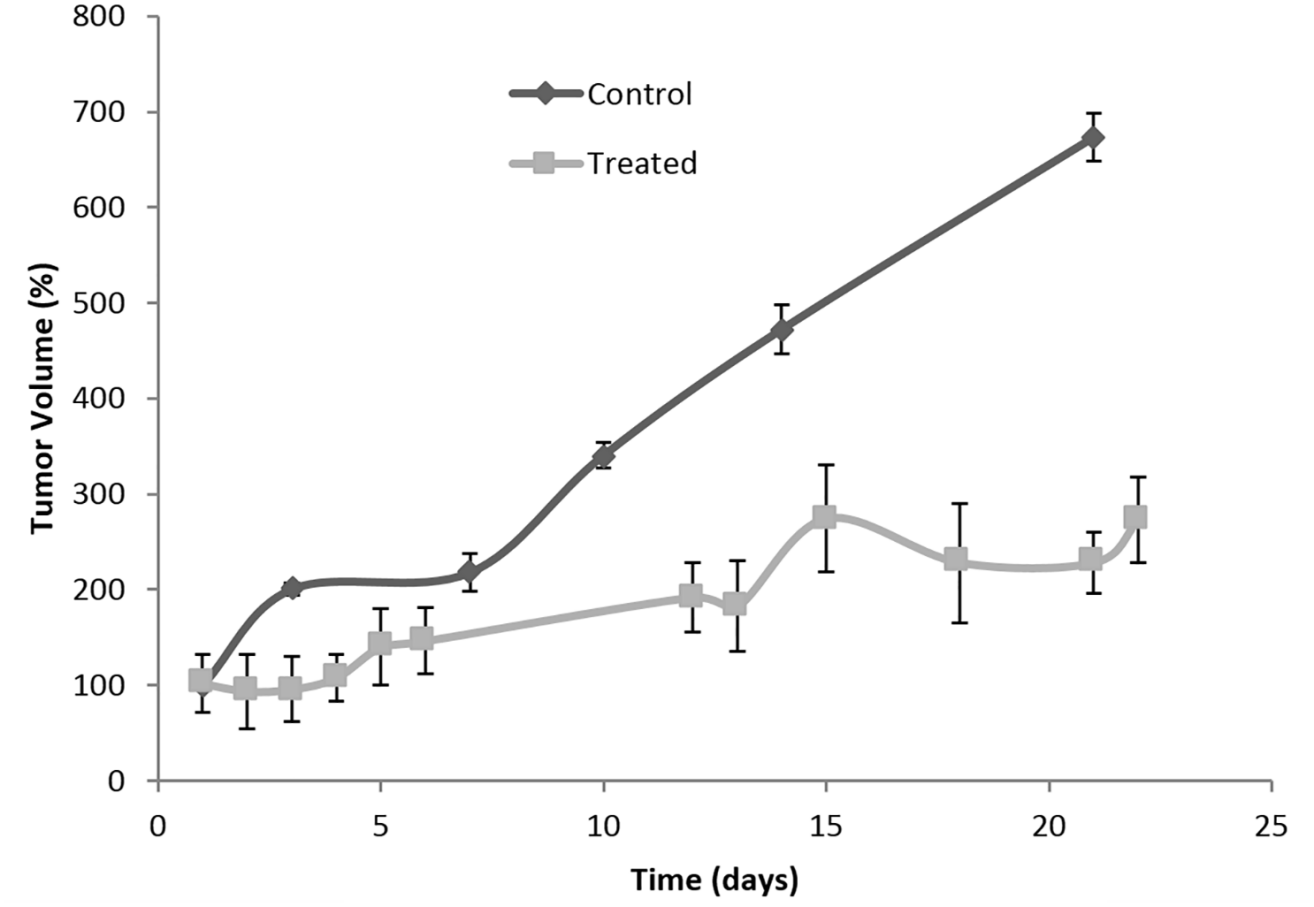
Percentage tumor volume growth after topical iontophoretic application of ZnPcS_4_ for 30 min followed by light irradiation (660 nm, 100 J/cm^2^). The treatment was performed at day 0 and 6. The results were expressed as the average ± SD, n = 3 for positive control—non-treated tumors; n = 5 for treated animals. Tumor volume growth was significantly decreased in treated animals (intercept of two linear regressions, F = 47.87 and p < 0.0001).

Fig 5 shows tumor growth as a function of time, monitored after the first 30 min of iontophoretic treatment followed by irradiation (660 nm, 100 J/cm^2^).

**Fig 6:**
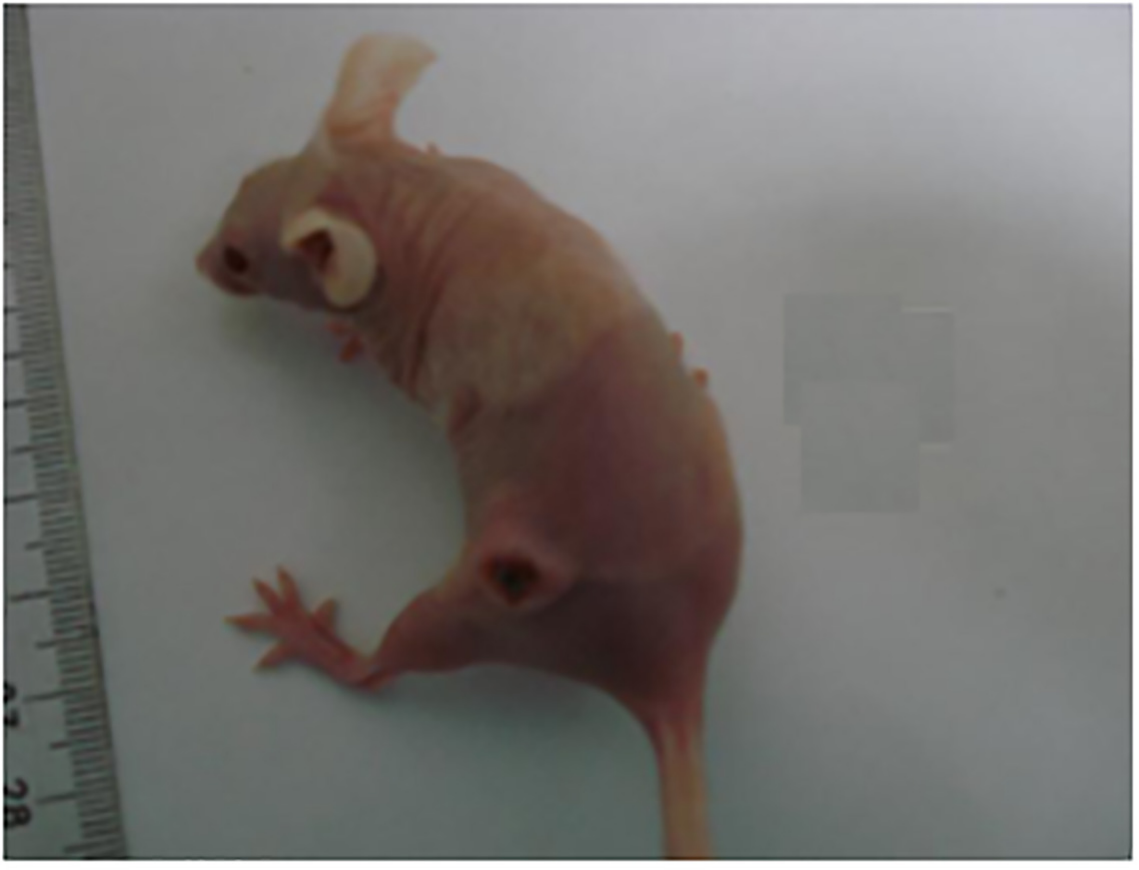
Photograph of necrosed tumor after PDT iontophoretic treatment.

Studies have shown that SCC A431 untreated tumors grow with time [29]. According to our findings, iontophoretic-treated tumors followed by PDT decreased the rate of tumor growth (Fig 5). Moreover, after the second stage of the treatment, tissue necrosis was observed in most animals (Fig 6). After the first stage however, no morphological alteration of the tumor was noticed macroscopically. It is interesting to emphasize that one animal, likely due to an error in the tumor cells injection, developed two individual tumors and only one was treated. After 18 days, the animal was sacrificed due to the accelerated growth of the untreated tumor, which compromised the vital functions of the animal.

It is known that ZnPcS_4_ absorbs photons after its exposure to light and initiates a photochemical reaction producing highly reactive oxygen species (^1^O_2_) which are responsible for the death of the tissue under treatment [31]. Our results suggest that the application of iontophoresis for 30 min allowed ZnPcS_4_ to penetrate to tumoral environment while the subsequent exposure to PDT (laser at 660 nm and 100 J/cm^2^) promoted tissue necrosis observed macroscopically. PDT may cause tumor cell death by apoptosis and/or necrosis when photosensitizers accumulate in the cell membranes or in the lysosomes [32]; this tumor cell death varies according to the cell type, oxygen level, photosensitizer concentration and light dose [33]. In addition to being essential for PDT, tissue accumulation of photosensitizers increases the animal immune response, thus increasing the destruction of the tumor cells [33].

Aggressive head and neck cancers usually begin in the squamous cells and have a microinvasive histological pattern. Surgery, radiation therapy or chemotherapy are usually combined to overcome these tumors. Based on our results, the association of iontophoresis with PDT could be an alternative that could be used concurrently with conventional treatment methods, thereby exerting a synergistic effect. Iontophoresis made possible the direct administration of a photosensitizer, instead of a precursor which is poorly converted into the photosensitizer in highly undifferentiated tumors [8]. Moreover, iontophoresis increases the photosensitizer penetration into deeper tumor regions and improves drug distribution in the tumors compared with passive administration.

In this work, ZnPcS_4_ was used as a model hydrophilic photosensitizer; however, other photosensitizers that have shown to benefit from iontophoresis for skin penetration, such as porphyrins or their precursors [7; 21], could be combined with iontophoresis for treatment of SCC or other solid tumor, as long as the tumor is accessible for the application of iontophoresis and the likes, as in the case of bladder cancers. Other points that should be taken into consideration in the application of a iontophoretic/PDT treatment is the need for drug formulation to be hydrophilic and allow the passage of the electric current; decrease of competitive ions in the formulation is also important to improve photosensitizer delivery. Moreover, it is important to determine the application time, which must be sufficient for drug penetration into the tumor but not through the tumor. Of course laser power control and the specific wavelength of the excitation source in PDT are important parameters to be controlled.

Thus, iontophoresis as a physical technique to increase photosensitizer permeation may be able to improve the efficiency of PDT in SCC treatment including more invasive ones.

## Conclusion

In summary, iontophoresis was a good strategy to improve ZnPcS_4_ penetration into skin tumors. PDT performed immediately after iontophoresis administration prevented the exponential growth of SCC tumors and caused necrosis of the tumor tissue. Our results show that iontophoresis targeted the photosensitizer to tumor cells by an adequate selection of electric current application time and formulation. We conclude that iontophoresis could be a potentially useful strategy for a non-invasive and more selective PDT.
